# How to Cure Some Cases of Acute Eczema

**Published:** 1885-08

**Authors:** Q. C. Smith

**Affiliations:** Austin, Texas


### HOW TO CURE SOME CASES OF ACUTE ECZEMA.

*By Q. C. Smith, M. D., Austin, Texas.*

For Daniel's Medical Journal

BATHE the affected parts for half an hour in Packer's Tar soap suds, *hot* as can be borne, reapplying the soap gently, but thoroughly, with a soft brush or mop, several times during the bathing process; then rinse off the soap with clean water, *hot* as can be borne, dry the parts very gently, then apply to the parts, with a soft mop, the folowing embrocation:

| | | |
|---|---|---|
| ℞ | Oleate Copper, | ʒi, |
| | Oleate Bismuth, | |
| | Oil Camphor, | |
| | Oil Sassafras *a a*, | ʒii, |
| | Carbolic Acid, (pure) | ʒss, |
| | Balsam Peru, | ℥ss, |
| | Castor Oil qs. ft., | ℥iv. |

Mix well. S. Apply once or twice a day, as directed.

Should the foregoing embrocation cause severe, continued smarting, it should be further diluted with good, fresh castor oil. For small children it should always be diluted to such a degree as that no smarting will result from its application. In case of severe presistent pruritus the embrocation should be applied, as before directed, twice a day, but usually once a day —at bedtime—is sufficient, provided the parts be well protected from the air and from chaping.

Thorough constitutional treatment should be promptly instituted and presistently carried out, in every case. As a constitutional remedy, the following formula is a favorite with us :

| | | |
|---|---|---|
| ℞ | Fl. Ex. Burdock Seed, | ʒii, |
| | Fowler's Solution, | ʒii, |
| | Syr. Ipecac, | |
| | Tinct. Aloes *a a*, | ℥ss, |
| | Comp. Syr. Stillingia qs, ft., | ℥iv. |

M. ft. Sol. S. Teaspoonful three times a day, just after meals, to be continued until the patient is cured and one or two weeks longer.

